# Nationwide Implementation of Hello World: A Dutch Email-Based Health Promotion Program for Pregnant Women

**DOI:** 10.2196/jmir.1183

**Published:** 2009-07-30

**Authors:** Mariska Bot, Ivon EJ Milder, Wanda JE Bemelmans

**Affiliations:** ^1^National Institute for Public Health and the EnvironmentBilthoventhe Netherlands

**Keywords:** Health promotion, Internet, pregnancy, email

## Abstract

**Background:**

In November 2006, an email-based health promotion program for pregnant women was implemented nationally in the Netherlands. The program consisted of emails containing quizzes with pregnancy-related questions tailored to the number of weeks of pregnancy. Emails were sent out once every 4 weeks, up to a maximum of nine emails.

**Objectives:**

The aims of the study were (1) to assess the recruitment of participants and their representativeness of the Dutch population and (2) to study differences in recruitment, program use, and program appreciation among women with different levels of education.

**Methods:**

Data from 13,946 pregnant women who enrolled during the first year of the program were included. Upon registration, participants were asked how they found out about the program and subsequently received an email questionnaire to assess demographic, lifestyle, and Internet characteristics. Program use was tracked, and participants were classified into five user groups (inactive to very active). Program appreciation (low, intermediate, and high) was assessed twice with an email questionnaire that was sent after the woman had received her third and sixth quiz email. Information about pregnant women and their characteristics was obtained from Dutch registries to assess representativeness of the study population.

**Results:**

About 8% of the pregnant women in the Netherlands enrolled in the program. Immigrants were underrepresented, and women with a low level of education seemed to be slightly underrepresented. Most women knew about the program from a promotional email sent by the organization (32%), followed by the Internet (22%) and midwives (16%). Women with little education were more often inactive users of the program than were highly educated women (15% vs 11%, *P* < .001), whereas highly educated women were more often very active users compared with women with little education (25% vs 20%, *P*< .001). However, women with less education were more likely than women with more education to have a high appreciation of the program after receiving three quiz emails (52% vs 44%, *P =* .001).

**Conclusions:**

In this real-life setting, pregnant women can be reached through an email-based health promotion program. Selective engagement by education level remains a challenge.

## Introduction

Pregnancy is a time when women are more conscious of health issues than during any other period in their life [1]. This may be a time when behavior can be positively changed, to have a long-term effect on the health of the mother, child, and family [2]. Nowadays, most women seek information about their pregnancy on the Internet [3-6], particularly for first-time pregnancies [4]. However, online medical information varies in quality and is often incomplete [7].

In 2006, the Dutch Ministry of Health initiated Hello World, an innovative email-based program for pregnant women in which reliable information about a healthy pregnancy is brought together into one health promotion program. The program was tested in a pilot study in Amsterdam [8,9] and was then improved and implemented nationwide in the Netherlands. The aim of the program was to provide comprehensive health information for pregnant women. Although knowledge on its own is criticized as being insufficient for behavior change, knowledge is a prerequisite for behavior change [10].

To the best of our knowledge, Hello World is the first eHealth program aimed at pregnant women that was implemented nationally. We are aware of only one previous study of an eHealth program for pregnant women conducted locally in Taiwan [11], which was aimed at promoting breastfeeding.

To be effective as a health promotion program, it is essential to reach the target group. Thus, efficacious recruitment strategies are needed. Several studies have investigated which recruitment strategies for Internet-based eHealth promotion programs result in high participation, but results were mixed [12-18]. In addition to enrollment, engagement is an important condition for efficacy since exposure to the health information is necessary. Unfortunately, the high attrition rate of Internet-based health promotion interventions is still an issue [19,20]. Both enrollment and engagement in health communication programs are often selective, favoring participation of relatively highly educated persons with a healthy lifestyle [19-21]. Indeed, the pilot study of Hello World showed that highly educated women not only enrolled in the program more often, but also used the program more intensively and longer than less-educated women [8].

The present study analyzes data of participants who enrolled during the first year of the nationwide-implemented program. The main purpose of the study was to describe the participants, their representativeness of all pregnant women in the Netherlands, and how they were recruited in a real-life setting. Our second aim was to compare differences in recruitment channels, program use, and program appreciation among women with varying levels of education.

## Methods

### Intervention

The program started on November 13, 2006, and is still available online. The present study analyzes data of pregnant women who enrolled in the first year. The program consisted of emails containing quizzes with pregnancy-related questions that were tailored to the number of weeks of pregnancy. Emails were sent once every 4 weeks. The first quiz email was sent at 8 weeks of pregnancy and the last one at 40 weeks. Women could enroll in the program at no cost anytime during their pregnancy and only received the quizzes that were applicable to their stage of pregnancy. Each quiz consisted of one question on each of the following six topics: nutrition, smoking, physical activity, safety, lifestyle/care, and pregnancy. Women could complete the quiz after receiving an email that contained one of the six questions as an example and a hyperlink to the entire quiz. Each question had two answer possibilities. Selecting an answer was followed by an automatic message on the website informing the women whether their answer was correct, an explanation of the correct answer, and, in most cases, a practical tip and a hyperlink to a related website providing additional or more detailed information. Several national health promotion institutes and the national platform of midwives were responsible for the content of the topic relevant to their expertise. Furthermore, women could ask online experts pregnancy-related questions.

**Figure 1:**
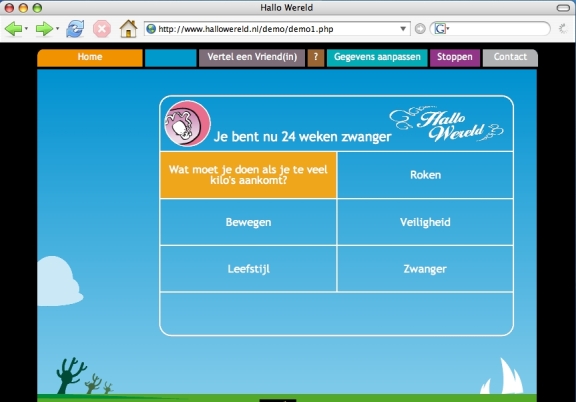
Screenshot of the quiz for week 24

The program was specifically developed to reach women with basic literacy skills, including the use of short text blocks and plain language. Figure 1 shows an example of a quiz question. For an overview of all quiz questions, translated into English, see the Multimedia Appendix.

### Study Participants

Pregnant women signed up for participation in Hello World on the website. Those who subscribed between the start of the program and November 13, 2007, were included in the study (n = 14,154). For the women who registered more than once, the most active account or the most recent inactive account was included; all other double accounts were excluded (n = 127). Additionally, data of nonpregnant persons and test users (n = 19) or those with an unlikely number of weeks of pregnancy (less then 0 or more than 40 weeks; n = 62) were excluded. Data of 13,946 participants were used for the analyses. Women who mentioned miscarriage as the reason for unsubscribing (n = 260) were included for assessment of enrollment, representativeness, and recruitment, but they were excluded from the analysis of program use and appreciation. The study was carried out in accordance with Dutch privacy legislation. Informed consent was obtained for all participants via the user conditions of the website that participants had to agree to when registering for Hello World. It was explained to the participants that answering the research questionnaires was voluntary and not a prerequisite for (further) use of the website.

### Recruitment

Several recruitment strategies were used to promote the program. From January 2007 onward, 50,000 Hello World leaflets were included in pregnancy present boxes, which could be requested by pregnant women on another pregnancy-related website. In addition, between January and November 2007, a promotional email was sent by the organization who implemented the intervention to almost 76,000 women who had requested a pregnancy present box. Furthermore, banners were placed on several websites, and advertisements for Hello World appeared when performing pregnancy-related searches in Google. As well, a total of 45,000 Hello World flyers were distributed in the majority of Dutch midwifery practices. In addition, recruitment took place through traditional mass media as advertisements in magazines and through an information stand at a national pregnancy fair.

### Measures

#### Demographic, Lifestyle, and Internet Characteristics

Upon enrollment, the women were asked to enter their first name, date of birth, email address, expected date of delivery, and how they found out about the website. In this paper, the last variable will be referred to as the “recruitment channel,” and it was categorized into the following eight types: midwife, email, Internet, pregnancy present box, pregnancy fair, peers, media (radio, television, magazines, newspapers), and other/unknown. Subsequent to enrollment, women received a baseline questionnaire by email to assess demographic, lifestyle, and Internet characteristics. Age was calculated from the date of birth. Ages < 15 and > 45 years were regarded as errors and were therefore not included in the age description. The number of weeks of pregnancy (0-14 weeks, 15-27 weeks, and 28-40 weeks) was calculated from the date of enrollment and the expected delivery date, assuming a pregnancy duration of 40 weeks.

The education level of the women was assessed as the highest level completed and was categorized as low (lower secondary education or less), intermediate (higher secondary education), or high (college or university) [22]. Women were regarded as immigrants when at least one of their parents was born outside the Netherlands [22]. If the woman herself was also born outside the Netherlands, she was considered a first-generation immigrant. Furthermore, immigrant women were divided into immigrants from Western counties and non-Western countries based on their parents’ country of birth. Information on parity, job participation, overweight before pregnancy, smoking status, and alcohol consumption was also obtained with the questionnaire.

Sufficient fruit and vegetable intake was based on meeting the Dutch guidelines for minimum intake levels of fruit and vegetables (ie, two pieces of fruit or one piece of fruit and one glass of fruit juice per day, and 150-200 g of vegetables per day) [23]. Sufficient physical activity was defined as being moderately active for at least 30 minutes on five or more days per week, according to the Dutch recommendations for physical activity [24]. No folic acid intake was defined as not currently using or not having used folic acid tablets. The questionnaire also included questions about Internet availability at home, time spent online per week (< 1 hour, 1-2 hours, and > 2 hours), and the use of the Internet for obtaining information about pregnancy, health, and/or parenting.

#### Program Use

Program use was registered continuously and included the quiz emails sent to the participant and the quiz questions accessed by the participant. The quiz emails contained a personal identification code to track the use of the quiz questions. All user data up to the end of August 2008 were included, thereby covering a whole pregnancy period after the last moment of registration in this study (November 13, 2007) to ensure that every woman could have received all quiz emails. If at least one of the quiz questions was opened by the participant, that woman was defined as a “user” of the quiz for that specific week. To describe program use, only the women who enrolled before or during week 28 of their pregnancy were included since they could have received a substantial number of quiz emails.

Program users were classified into five categories: (1) inactive (did not use any of the received quizzes), (2) ceased (did not use the last two received quizzes or unsubscribed themselves from the program after using one or more quizzes), (3) moderately active (used at least two of the last quizzes, but less than 2/3 of all quizzes), (4) active (used at least two of the last quizzes, and at least 2/3 of all quizzes), and (5) very active (used all quizzes). The quiz email for week 40 was not taken into account since we expected that not all pregnant women would be able to open quiz emails around the time of delivery. Indeed, the data showed that this quiz was completed less often than other quizzes.

#### Program Appreciation

Appreciation of the program was assessed by means of an online self-administered questionnaire that was sent in the week after the participants received their third quiz email and their sixth quiz email. As women could enroll themselves anytime throughout their pregnancy, timing of the questionnaire varied among participants. The same questionnaire was used for both evaluations and included eight positively formulated statements about the program that could be rated on a five-point Likert scale (“totally agree” to “totally disagree”). Examples of the statements include “the information on Hello World is reliable” and “the information on Hello World is recent.” Based on the number of statements for which the response was “agree” or “totally agree,” program appreciation was categorized into low (agreed with 0-4 statements), intermediate (agreed with 5-6 statements), or high appreciation (agreed with 7-8 statements).

### Data Analyses

Data analyses were performed in 2008. Response rates of the questionnaire were based on the number of questionnaires sent to the participants and the number of questionnaires completed by the participants. The demographic characteristics of the pregnant women participating in the email-based health promotion program were described and compared with existing data of pregnant women in the Netherlands [22]. Because younger women may not have completed their education, age-stratified education levels were compared. Demographic, lifestyle, and Internet characteristics; recruitment channel; program use; and program appreciation were compared among participants with different levels of education. For program use and education level, additional analyses were conducted after stratification for parity.

For all analyses, differences between education levels were tested with chi-square tests for categorical variables and analysis of variance for continuous variables.

Because education level was associated with several characteristics and lifestyle factors, the associations between education level and program use and program appreciation, after adjustment for these characteristics, were tested using ordinal logistic regression. The final multivariate models were constructed by including all variables that were significantly associated with the independent variable (program use or appreciation) in the bivariate models (including education level and one other characteristic) or the multivariate model (including all characteristics). Statistical significance was set at *P* < .05. All data analyses were carried out using SAS for Windows, version 9.1 (SAS Institute, Cary, NC, USA).

## Results

### Representativeness of the Participants

Based on the number of enrolled women in the program (n = 13,946) and the number of living newborns in the Netherlands in 2007 (n = 18,1336), we estimated that approximately 8% of all pregnant women in the Netherlands enrolled in the Hello World program (Table 1). Most participants (9210/12,802, 72%) in our study were aged 25-35 years. Although there were fewer low-educated women in the program than in the Netherlands for the 15-25 and 35-45 year age groups, the percentage of low-educated women among the 25-35 year age group was comparable to that of the Netherlands. In our study, the percentage of immigrants (1583/8833, 18%) was smaller than the overall proportion found in the Netherlands (24%). The majority of participants (5568/8833, 63%) were pregnant with their first child, which was higher than the percentage of first-born babies in the Netherlands.

**Table 1:** Characteristics of the participants of Hello World

Characteristic		Hello World	The Netherlands^a^
Number of pregnant women		13,946	18,1336^b^
		Mean ± SD	Mean
Age of those pregnant with their first child (years)^c^		28.3 ± 4.4	29.4^d,e^
Age at enrollment (years)^c^		29 ± 4.5	31.1^d,f^
		No. (%)	%
Response rate for baseline questionnaire^g^		8833/13,942 (63)	–
Pregnant with first child		5568 (63)	45^h^
Age group			
15-25 years		2368 (18)	10^d^
25-35 years		9210 (72)	64^d^
35-45 years		1224 (10)	26^d^
Education level			
15-25 years	Low	594 (43)	50^i^
	Intermediate	660 (47)	40^i^
	High	142 (10)	10^i^
25-35 years	Low	1032 (17)	17^i^
	Intermediate	2366 (40)	46^i^
	High	2544 (43)	37^i^
35-45 years	Low	154 (19)	25^i^
	Intermediate	260 (33)	47^i^
	High	386 (48)	28^i^
Employment			
Full-time job (≥ 32 hours/week)		4120 (47)	NA^j^
Part-time job (< 32 hours/week		3058 (35)	NA^j^
No job		1619 (18)	24^k^
Immigrant		1583 (18)	24^k^
First generation		635 (7)	14^k^
Non-Western		753 (9)	14^k^

^a^Data from Statistics Netherlands [22].

^b^Number of living newborns in the Netherlands in 2007.

^c^Only those aged ≥ 15 and ≤ 45 years were included. For Hello World participants, age at enrollment included 12,802 women and age of those pregnant of their first child included 5252 women.

^d^Only pregnant women.

^e^ Mean age of those pregnant with their first child in 2007.

^f^Mean age of women at the time of delivery in 2007.

^g^Proportion based on the number of returned questionnaires and the number of questionnaires sent to the women.

^i^Including pregnant and nonpregnant women.

^j^ Not available.

^k^Including pregnant and nonpregnant women aged 15-45 years.

### Recruitment Channel

As shown in Table 2, the most reported recruitment channel was email (32%), followed by the Internet (22%) and midwives (16%). This order was observed for all education levels. However, relatively more women with lower education mentioned email as the recruitment channel (38%) than did highly educated women (24%; χ^2^= 154.5, *P <* .001); the reverse was observed for midwives as the recruitment channel (14% vs 20% for low and high education, respectively; χ^2^ = 38.1, *P <* .001).

**Table 2:** Recruitment channel by education level

Recruitment Channel	Total^a^ (n = 13,946)		Low (n = 1940)		Intermediate (n = 3548)		High (n = 3317)	
	No.	%	No.	%	No.	%	No.	%
Email	4399	32	744	38	1280	36	804	24
Internet (surfing, Google, other websites)	3023	22	384	20	767	22	779	23
Midwife	2214	16	279	14	560	16	674	20
Pregnancy present box	1116	8	164	8	285	8	303	9
Peers	1041	7	135	7	229	6	220	7
Media (radio, television, magazine, newspaper)	653	5	76	4	146	4	197	6
Pregnancy fair	585	4	35	2	100	3	115	3
Other/unknown	915	7	123	6	181	5	225	7

^a^ The low, intermediate, and high education levels do not add to the total group because not all women completed the baseline questionnaire with the question about education.

### Characteristics by Education Level

Table 3 presents the characteristics of the pregnant women by education level. More highly educated women (2257/3312, 68%) were pregnant with their first child than were women with a low level of education (1115/1937, 58%; χ^2^ = 65.7, *P <* .001). Low-education women more often smoked during pregnancy, less often had sufficient fruit and vegetable intake, and less often used folic acid than high-education women. Almost all women had access to the Internet at home and used it for obtaining pregnancy-related information.

**Table 3:** Demographic, lifestyle, and Internet characteristics by education level^a^

	Low		Intermediate			High		χ^2b^	*P*^c^
	No.	%^b^	No.	%^b^		No.	%^b^		
Age (years)	1780	*27.5(5.2)^d^*	3286	*28.8 (4.3)^d^*		3072	*30.7 (3.5)^d^*	*351.0^e^*	< .001
**Pregnancy**									
Pregnant with first child	1115/1937	*58*	2179/3544		*61*	2257/3312	*68*	*65.7*	< .001
Number of weeks of pregnancy at enrollment	1940	*18.4 (9.2)^d^*	3548		*18.0 (9.3)^d^*	3317	*17.4 (9.1)^d^*	*8.1^e^*	< .001
1st trimester	795/1940	*41*	1510/3548		*43*	1481/3317	*45*	*15.2*	.004
2nd trimester	783/1940	*40*	1394/3548		*39*	1326/3317	*40*		
3rd trimester	362/1940	*19*	644/3548		*18*	510/3317	*15*		
**Lifestyle characteristics**									
Overweight before pregnancy (BMI ≥ 25 kg/m^2^) ^f^	752/1913	*39*	1297/3495		*37*	845/3280	*26*	*137.8*	< .001
Smoking								*906.1*	< .001
Smoker	465/1938	*24*	329/3543		*9*	57/3317	*2*		
Cessation because of pregnancy	443/1938	*23*	666/3543		*19*	399/3317	*12*		
Nonsmoker	1030/1938	*53*	2548/3543		*72*	2861/3317	*86*		
Alcohol use during pregnancy	41/1934	*2*	87/3538		*2*	142/3308	*4*	*26.9*	< .001
Sufficient vegetable intake^g^	253/1225	*21*	518/2263		*23*	674/2106	*32*	*69.2*	< .001
Sufficient fruit intake^g^	482/1266	*38*	1037/2337		*44*	1133/2159	*52*	*71.0*	< .001
Sufficient physical activity^h^	553/1325	*42*	885/2393		*37*	819/2239	*37*	*10.8*	.005
No folic acid used	304/1932	*16*	287/3537		*8*	141/3308	*4*	*210.4*	< .001
**Internet characteristics**									
Internet at home	1847/1930	*96*	3428/3544		*97*	3259/3309	*98*	*38.6*	< .001
Internet use								*36.4*	< .001
< 1 hour/week	222/1933	*11*	370/3540		*10*	261/3311	*8*		
1-2 hours/week	610/1933	*32*	1217/3540		*34*	1030/3311	*31*		
> 2 hours/week	1101/1933	*57*	1953/3540		*55*	2020/3311	*61*		
Use of Internet for information about pregnancy	1654/1932	*86*	3176/3542		*90*	2987/3310	*90*	*29.5*	< .001

^a^ Due to missing values, the totals in each column vary. Italics represents significant results.

^b^ Unless otherwise noted.

^c^ The chi-square test was used for categorical variables, and analysis of variance was used for continuous variables.

^d^ Mean (SD).

^e^ F-value.

^f^ Body mass index (BMI) was calculated as the self-reported weight before pregnancy (kg) and height (m^2^).

^g^ Meeting the current Dutch guidelines for sufficient fruit and vegetable intake. Sufficient fruit intake: at least two pieces of fruit per day or one piece supplemented by fruit juice. Sufficient vegetable intake: at least 150-200 g of vegetables per day.

^h^ At least 30 minutes of being moderately active on five or more days per week.

### Program Use

For the analyses of program use, we included only the women who enrolled in week 28 of pregnancy or earlier (11,415/13,686; 83%). On average, the participants received a mean of 5.6 ± 1.8 quiz emails and opened 2.8 ± 2.3 of them to complete at least one of the six questions. Women completed, on average, 3.8 ± 1.6 of the six available questions for each opened quiz.

**Figure 2:**
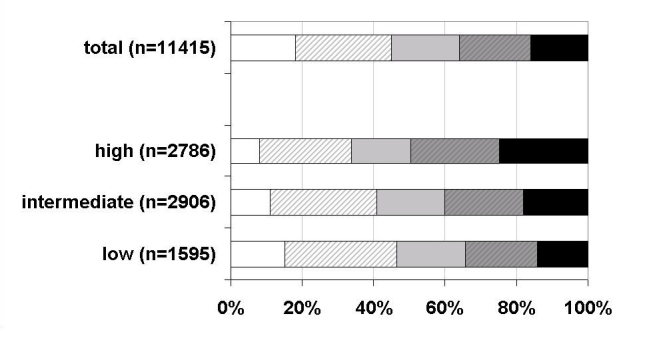
Type of program user by education level

Figure 2 shows the distribution of program users for all women who registered in week 28 of pregnancy or earlier. Program users were classified into five categories: (1) inactive (did not use any of the received quizzes), (2) ceased (did not use the last two received quizzes or unsubscribed themselves from the program after using one or more quizzes), (3) moderately active (used at least two of the last quizzes, but less than 2/3 of all quizzes), (4) active (used at least two of the last quizzes, and at least 2/3 of all quizzes), and (5) very active (used all quizzes). The quiz email of week 40 was not taken into account, and miscarriages were excluded. In Figure 2, the numbers of women with low, intermediate, and high education do not add to the total group because not all women completed the baseline questionnaire with the question about education level.

About 18% (2075/11,415) of the women never opened a quiz email, 27% (n = 5166) ceased participating, and 16% (n = 1849) opened all quiz emails received. Inactivity was observed more among the women with low education (15%, 242/1595) than among the women with high education (8%, 220/2786; χ^2^ = 56.7, *P <* .001). Similarly, ceased participation was more common among low-education women (31%, 497/1595) than among high-education women (26%, 712/2786; χ^2^ = 20.5, *P <* .001).

Very active users were more likely to be women with a high level of education (25%, 710/2786) than with a low level of education (14%, 229/1595; χ^2^ = 93.7, *P <* .001). When repeating these analyses stratified for those pregnant for the first time and those not, comparable results for education level were found (results not shown).

Because education level was associated with program use as well as with several lifestyle factors (see Table 3), we tested the independent association of education level and program use using multivariate ordinal regression. In the multivariate model, higher education, being older, being pregnant with the first child, number of weeks of pregnancy, being a nonsmoker, and not being overweight were independently associated with program use, whereas alcohol use and folic acid use were not.

### Program Appreciation

The first appreciation questionnaire was completed by 3763/12,201 women who received it (response rate 31%); the second appreciation questionnaire was completed by 1926/8666 women who received it (response rate 22%). Half (n = 1840) of the 3763 women who answered the first questionnaire had a high appreciation of the program, more than one quarter (n = 1091) showed an intermediate appreciation, and less than one quarter (n = 832) had a low appreciation. Low-education women were more likely to show a high appreciation (52%, 307/593) of the program than high-education women (44% 539/1219; χ^2^ = 17.9, *P =* .001) in the first appreciation questionnaire. A similar pattern was seen for the second appreciation questionnaire (data not shown).

Because education level was associated with program appreciation as well as with several lifestyle factors (see Table 3), we tested the independent association of education level and program appreciation using multivariate ordinal regression. In the multivariate model, being younger, being pregnant with the first child, and not using alcohol during pregnancy were independently associated with higher program appreciation, while education level and overweight were not.

## Discussion

Our study describes the enrollment and engagement rates in a national email-based health education program aimed at pregnant women in a real-life setting. We estimate that about 8% of all pregnant women in the Netherlands enrolled in the Hello World program. Women with less education seemed to be slightly underrepresented, and immigrants seemed to be underrepresented compared to the Dutch female population aged 15-45 years. In particular, women who were pregnant with their first child enrolled in the program. Most women cited email as their recruitment channel, followed by the Internet and midwives. In agreement with results of the pilot study [8], program use differed between women with low and high levels of education. Low-education women were more satisfied with the program than high-education women.

To our knowledge, Hello World is the first national online health education program aimed at pregnant women. The program incorporates several elements that have shown to be effective in online health education programs. These elements include tailored messages, which are known to be appreciated by pregnant women [3]. In addition, emails were sent every 4 weeks to inform the women about the availability of new quizzes, since previous studies showed that email reminders have a positive effect on the number of website revisits and were suggested as a strategy to retain participants [16,25]. Furthermore, a combination of online as well as offline recruitment strategies were used so that women who used the Internet less often could also find out about the program.

In the Netherlands, the majority of pregnant women visit midwives as the first line of care during pregnancy [26]. One of the tasks of midwives is to provide information about a healthy pregnancy. Thus, this is a natural setting for health promotion and interventions. A few studies have evaluated the effectiveness of interventions (eg, breastfeeding and smoking cessation) but with mixed results [27,28]. To the best of our knowledge, none of these interventions have been implemented nationally.

### Strengths and Limitations

Strengths of our study were its large sample size and real-life setting. In addition, program use was registered objectively and was not self-reported.

Some limitations need to be stressed. The response rate for the questionnaires dropped from 63% for the baseline questionnaire to 22% for the second appreciation questionnaire. Additional analyses showed that lower response rates on the questionnaires were related to low education level, younger age, and nonactive program use. Further, participation rates could not be calculated for the different recruitment strategies. In addition, it is likely that not all preterm births and miscarriages were reported. Since delivery and miscarriage will probably result in cessation of program use, this may have led to an underestimation of program use in pregnant women.

We estimated that 8% of all pregnant women in the Netherlands enrolled in the Hello World program. Our estimation has a few drawbacks. First, it is based on the number of living newborns in the Netherlands in 2007, thereby not taking miscarriages into account. However, as most miscarriages take place early in pregnancy and the women enrolled at 18 weeks of pregnancy on average, the number of miscarriages probably was small. Second, we cannot exclude that some women registered twice using different email addresses. In addition, nonpregnant persons or Dutch-speaking women living abroad may also register for the program. This may have led to an overestimation of the enrollment rate in our study. Third, our enrollment rate was based on all pregnant women, including those who were not exposed to the intervention. This must be kept in mind when comparing our enrollment rate with other studies. For example, the pilot study showed an enrollment rate of 17% [8], but this rate was based on the number of pregnant women who were invited to participate by midwives. Since only a fraction of all pregnant women probably knew about the program in our study, our enrollment rate appears reasonable.

We had to rely on existing data for describing the representativeness of the participants. In the pilot study, selective enrollment was observed, favoring enrollment of higher-educated women [8]. In contrast, in this study, the percentage of women with a low level of education was comparable to that in the Dutch female population aged 25-35 years. Almost three quarters of the women enrolled in Hello World belong to this age group. However, women with less education have more children than higher-educated women in the Netherlands [29] and may therefore be slightly underrepresented. Pregnant women with a low level of education need more attention since previous studies have shown that these women more often smoked during pregnancy [30], less often breastfed their children up to 6 months after delivery [31], and less often were aware of the benefits of folic acid and used folic acid less often during pregnancy [32,33]. Our study confirmed less favorable health behaviors in lower-educated women compared to higher-educated women, except for alcohol consumption and physical activity. The large number of women pregnant with their first child is desirable since they have the highest need for pregnancy-related information [3].

In our study, more participants were recruited by online strategies than by offline strategies. Similar results were found by Feil et al [14] and Thüring et al [13]. Further, in our study, lower-educated women were more often recruited by email than were highly educated women, whereas the reverse was observed for recruitment by midwives. It is possible that these differences can be partly explained by differences in exposure.

Eysenbach [34] has discussed the importance of studying attrition in Internet-based interventions. High dropout rates in Internet interventions are common, and selective dropout is frequently observed [20,34]. In our study, one sixth of the participants never opened a quiz email, and more than a quarter stopped participating. We found a higher level of program use among highly educated pregnant women compared to lower-educated pregnant women. This is similar to the pilot study [8] but is in contrast to Verheijden et al [20], who found no differences between education level for the usage of an online health promotion program in a subsample of the general Dutch population. Although the level of engagement was lower among low-education women, these women appreciated the program more than highly educated women. It is possible that the program was too easy and concise for highly educated women and that they therefore appreciated the program less.

### Future Work

We have measured both enrollment and engagement, which are important prerequisites for exposure to health messages in the program. However, the effect of these messages on health outcomes is unknown [3] and should be investigated in randomized controlled trials. More research is needed on the differences of processing health information among persons with varying levels of education. Effective methods for increasing enrollment and engagement need to be further studied.

### Conclusions

In conclusion, this study showed that about 8% of the pregnant women in the Netherlands could be reached through an Internet-based health promotion program. Lower-educated women seemed to be slightly underrepresented. Lower-educated women were less actively engaged than highly educated women but appreciated the program more. Most women were recruited by online recruitment strategies rather than traditional channels. Reducing selective attrition remains a challenge in Web-delivered health promotion programs.

## Multimedia Appendix

Overview of quiz questions

## References

[1] Anderson AS (2001). Symposium on 'nutritional adaptation to pregnancy and lactation'. Pregnancy as a time for dietary change?. Proc Nutr Soc.

[2] Verbeke Wim, De Bourdeaudhuij Ilse (2007). Dietary behaviour of pregnant versus non-pregnant women. Appetite.

[3] Bernhardt Jay M, Felter Elizabeth M (2004). Online pediatric information seeking among mothers of young children: results from a qualitative study using focus groups. J Med Internet Res.

[4] Szwajcer EM, Hiddink GJ, Koelen MA, van Woerkum CM (2005). Nutrition-related information-seeking behaviours before and throughout the course of pregnancy: consequences for nutrition communication. Eur J Clin Nutr.

[5] Larsson Margareta (2009). A descriptive study of the use of the Internet by women seeking pregnancy-related information. Midwifery.

[6] Shaw E, Howard M, Chan D, Waters H, Kaczorowski J, Price D, Zazulak J (2008). Access to web-based personalized antenatal health records for pregnant women: a randomized controlled trial. J Obstet Gynaecol Can.

[7] Rand Health (Prepared for the California HealthCare Foundation) (2001). Proceed with Caution: A Report on the Quality of Health Information on the Internet. Complete Study.

[8] van Zutphen Moniek, Milder Ivon E, Bemelmans Wanda J (2008). Usage of an online healthy lifestyle program by pregnant women attending midwifery practices in Amsterdam. Prev Med.

[9] van Zutphen Moniek, Milder Ivon E, Bemelmans Wanda J (2009). Integrating an eHealth program for pregnant women in midwifery care: a feasibility study among midwives and program users. J Med Internet Res.

[10] Green L, Kreuter MW (1991). Health Promotion Planning: An Educational and Environmental Approach. 2nd edition.

[11] Huang Mei Zen, Kuo Su-Chen, Avery Melissa D, Chen Wei, Lin Kuan-Chia, Gau Meei-Ling (2007). Evaluating effects of a prenatal web-based breastfeeding education programme in Taiwan. J Clin Nurs.

[12] Martin-Diener Eva, Thüring Nicole Response to a single media announcement for an internet based hepa-intervention.

[13] Thüring Nicole, Martin-Diener Eva, Martin Brian Effectiveness of an interactive Internet program promoting physical activity: the feasibility of an Internet-based, randomized study design.

[14] Feil Edward G, Noell John, Lichtenstein Ed, Boles Shawn M, McKay H Garth (2003). Evaluation of an Internet-based smoking cessation program: lessons learned from a pilot study. Nicotine Tob Res.

[15] Koo Malcolm, Skinner Harvey (2005). Challenges of Internet Recruitment: A Case Study with Disappointing Results. J Med Internet Res.

[16] Spittaels Heleen, De Bourdeaudhuij Ilse (2006). Implementation of an online tailored physical activity intervention for adults in Belgium. Health Promot Int.

[17] Gordon Judith S, Akers Laura, Severson Herbert H, Danaher Brian G, Boles Shawn M (2006). Successful participant recruitment strategies for an online smokeless tobacco cessation program. Nicotine Tob Res.

[18] Franklin Patricia D, Rosenbaum Paula F, Carey Michael P, Roizen Michael F (2006). Using sequential e-mail messages to promote health behaviors: evidence of feasibility and reach in a worksite sample. J Med Internet Res.

[19] Glasgow Russell E, Nelson Candace C, Kearney Kathleen A, Reid Robert, Ritzwoller Debra P, Strecher Victor J, Couper Mick P, Green Beverly, Wildenhaus Kevin (2007). Reach, engagement, and retention in an Internet-based weight loss program in a multi-site randomized controlled trial. J Med Internet Res.

[20] Verheijden Marieke W, Jans Marielle P, Hildebrandt Vincent H, Hopman-Rock Marijke (2007). Rates and determinants of repeated participation in a web-based behavior change program for healthy body weight and healthy lifestyle. J Med Internet Res.

[21] Thoolen Bart, de Ridder Denise, Bensing Jozien, Gorter Kees, Rutten Guy (2007). Who participates in diabetes self-management interventions?: Issues of recruitment and retainment. Diabetes Educ.

[22] Statistics Netherlands. Voorburg/Heerlen.

[23] Health Council of the Netherlands (2006). Richtlijnen goede voeding 2006 [Guidelines on healthy nutrition 2006].

[24] Kemper HGC, Ooijendijk WTM, Stiggelbout M (2000). Consensus over de Nederlandse Norm voor Gezond Bewegen [Consensus about the Dutch recommendation for physical activity to promote health]. Tijdschr Soc Gezondheidsz.

[25] Leslie E, Marshall AL, Owen N, Bauman A (2005). Engagement and retention of participants in a physical activity website. Prev Med.

[26] Althuizen Ellen, van Poppel Mireille N, Seidell Jacob C, van der Wijden Carla, van Mechelen Willem (2006). Design of the New Life(style) study: a randomised controlled trial to optimise maternal weight development during pregnancy. [ISRCTN85313483]. BMC Public Health.

[27] Kools Els J, Thijs Carel, Kester Arnold D, van den Brandt Piet A, de Vries Hein (2005). A breast-feeding promotion and support program a randomized trial in The Netherlands. Prev Med.

[28] de Vries Hein, Bakker Martijntje, Mullen Patricia Dolan, van Breukelen Gerard (2006). The effects of smoking cessation counseling by midwives on Dutch pregnant women and their partners. Patient Educ Couns.

[29] van Agtmaal-Wobma E, van Huis M (2008). De relatie tussen vruchtbaarheid en opleidingsniveau van de vrouw [The relationship between fertility and educational level of the woman]. CBS Bevolkingstrends.

[30] Lu Ying, Tong Shilu, Oldenburg Brian (2001). Determinants of smoking and cessation during and after pregnancy. Health Promot Int.

[31] Flacking Renée, Nyqvist Kerstin Hedberg, Ewald Uwe (2007). Effects of socioeconomic status on breastfeeding duration in mothers of preterm and term infants. Eur J Public Health.

[32] de Walle HE, Cornel MC, de Jong-van den Berg LT (2002). Three years after the dutch folic acid campaign: growing socioeconomic differences. Prev Med.

[33] Timmermans S, Jaddoe VW, Mackenbach JP, Hofman A, Steegers-Theunissen RP, Steegers EA (2008). Determinants of folic acid use in early pregnancy in a multi-ethnic urban population in The Netherlands: The Generation R study. Prev Med.

[34] Eysenbach Gunther (2005). The law of attrition. J Med Internet Res.

