# Supplementary material for: Nationwide Implementation of Hello World: A Dutch Email-Based Health Promotion Program for Pregnant Women
Source: J Med Internet Res. 2009 Jul 30;11(3):e24. doi: 10.2196/jmir.1183 (PMC2763403; doi:10.2196/jmir.1183)
Supplement: Supplementary file 1 [file jmir_v11i3e24_app1.pdf]

Appendix 1: Overview of quiz questions

Weeks pregnant	Nutrition	Exercise	Lifestyle / Care	Smoking	Safety	Pregnancy
8	Can you do something about morning sickness?	Does it still make sense to train your straight abdominal muscles?	How much alcohol can you drink without putting your baby at risk?	Congratulations, you are pregnant! What can you do to give your baby a healthy start? (with respect to smoking)	Is it safe to eat all cheese products during pregnancy?	Can I have my baby tested for Down syndrome?
12	When can you stop taking folic acid?	When you already have a toddler, or when you have toddlers in your surroundings, is it wise to lift them?	Some women have to vomit when they are pregnant. Is it wise to brush your teeth after vomiting?	Why is it harmful to smoke during pregnancy?	Is it safe to eat raw fish and raw meat during your pregnancy?	Some women lose some blood at the beginning of their pregnancy. Does this mean they will have a miscarriage?
16	What can you do when you are constipated?	Does your baby already move?	After three months of pregnancy, you may drink an alcoholic drink occasionally..	Is it important to prepare yourself to quit smoking?	You want to buy a cod and a mattress for your baby. What are things you need to consider?	Can I visit the sauna when I am pregnant?
20	Do you have to eat more during pregnancy?	You are twenty weeks pregnant. Your belly is expanding. Is it healthy to exercise at this stage of pregnancy?	What is the best time to visit the dentist?	When you quit smoking, you will soon be free of your addiction.	You want to buy a pram. What are the things you need to consider?	Sometimes you can feel short, stabbing pain when you get up or when you turn over in bed. You feel this pain under in your belly. What are the causes of your pain?
24	What should you do when you gain too many kilograms?	Is it important to exercise when you are pregnant?	What happens to your baby when you take an alcoholic drink?	Two cigarettes a day want do any harm.	You want to buy a play pen. What are things you need to consider?	I have now a lot more vaginal discharge than I had before pregnancy. Is this normal?

28	Should you take extra vitamins?	You don't want to go to the gym, but you would like to get some exercise. Are there special exercise courses for pregnant women?	What is the influence of pregnancy on your teeth and gums?	What to do when people are smoking in your environment?	You are pregnant. Do you have to wear a seat belt?	When should you feel your baby move every day?
32	What can you do against heartburn?	Can you use your abdominal muscles until the end of your pregnancy when getting out of bed?	Can harm to the baby as a result of regular alcohol use be undone?	When you quit smoking, you may become stressed. What is better: to continue smoking or to quit and have stress?	Can my baby sleep under a duvet?	Can you decide yourself what the last name of your child will be?
36	What can you do about swollen hands and feet?	You can only get your painful muscles supple by massage.	Are dental diseases communicable?	Does it still make sense to quit smoking at this moment?	Have you already tried to put the baby car seat in the car?	When do you have to decide where you want to give birth?
40	Can you start dieting 6 weeks after delivery?	Can you start exercising immediately after pregnancy?	You can drink a glass of alcohol at the end of the pregnancy.	After delivery I can start smoking again, because it is not harmful for my baby anymore.	How should your baby sleep when it is recently born?	Can I breastfeed my baby immediately after delivery?
